# Anti-Oxidation and Anti-Inflammatory Potency Evaluation of Ferulic Acid Derivatives Obtained through Virtual Screening

**DOI:** 10.3390/ijms222111305

**Published:** 2021-10-20

**Authors:** Yuqi Shi, Xuelian Chen, Shaojia Qiang, Jie Su, Jiazhong Li

**Affiliations:** School of Pharmacy, Lanzhou University, 199 West Donggang Rd., Lanzhou 730000, China; yqshi19@lzu.edu.cn (Y.S.); xlchen2020@lzu.edu.cn (X.C.); qiangshj19@lzu.edu.cn (S.Q.); suj20@lzu.edu.cn (J.S.)

**Keywords:** ferulic acid, virtual screening, free radical scavenging, anti-inflammation, antioxidant

## Abstract

Various factors such as ultraviolet rays can cause a continuous threat to our skin, resulting in inflammation or oxidation problems. Ferulic acid (FA), with certain antioxidant and anti-inflammatory properties, is widely used in many cosmetics, even used to treat various diseases in the clinic. In this study, the FA structural skeleton was used to search for FA derivatives. Then, molecular docking, the rule of five, and Veber rules were performed to virtually screen compounds that can bind to proteins with a good drug likeness. DPPH and ABTS were used to evaluate their antioxidant potency and an MTT assay was employed to investigate the toxicities of the compounds, while Griess Reaction System and ELISA were used to judge the concentration variations of NO and different inflammatory factors (TNF-α, IL-1β, and IL-6). Western blotting featured nitric oxide synthase (iNOS) and cyclooxygenase-2 (COX-2) protein expression levels. The trend of the intracellular changes of reactive oxygen species (ROS) was detected by the DCFH-DA method and fluorescence staining. As a result, we found that the ferulic acid derivative S-52372 not only had certain scavenging effects on free radicals in biochemical experiments, but also prevented inflammation and oxidative stress in LPS-stimulated RAW264.7 cells in the cellular environment; intracellular ROS and inflammatory mediators, including iNOS, COX-2, TNF-α, and IL-6, were also suppressed. In a computer prediction, S-52372 owned better water solubility and lower toxicity than FA. This compound deserves further research to find an ideal FA derivative.

## 1. Introduction

In our daily life, external oxidative factors often promote the occurrence of skin inflammation, and then inflammation promotes the process of oxidative stress to a certain extent [1]. Oxidative stress and inflammation have a close relationship with modern lifestyle-related health problems, such as aging and cardiovascular diseases [2]. Persistent or chronic inflammation can affect the normal metabolic processes, cause the development and progression of diseases [3], and generate damage to the skin. Hence, in order to prevent and protect the skin from peroxidation and inflammation problems, it is meaningful to control excessive oxidative stress and inflammatory response.

Lipopolysaccharide (LPS) can stimulate the activation of macrophages, leading to an inflammatory response [4]. Therefore, LPS are frequently used as a common material to stimulate macrophages and to build an inflammation model that associates inflammatory responses in vitro [5]. During the whole process, after stimulation by LPS, a great amount of reactive oxygen species (ROS) is produced when excessive oxidative stress occurs in macrophages [6], and then there is a distraction between pro-inflammatory and anti-inflammatory factors [7], causing a gradual increase in the inflammatory response. Throughout the whole process, lasting and high-level expression of nitric oxide (NO), iNOS, COX-2, tumor necrosis factor (TNF)-α, and interleukin (IL)-6 occur in the overall condition of the body and finally leads to a disorder [8].

Ferulic acid (FA), a photoprotective agent and a natural product, is a polyphenolic compound commonly found in fruits, vegetables, and beverages [9]. It has been reported that FA owns the ability to scavenge free radicals and anti-oxidative and anti-inflammatory factors; hence, FA may be able to play a role in free radical-induced diseases [10] and is widely used in the pharmaceutical, food, and cosmetics industries [11]. Researchers have found that FA not only has a tendency to scavenge free radicals, but can also inhibit free radical generation [12]. Thus, ferulic acid has been wildly applied in skin caring by acting on the main skin structures such as keratinocytes, fibroblasts, collagen, and elastin to delay skin photoaging processes [13]. However, it is also reported that the stability of ferulic acid is affected by temperature, pH, and many other factors, which greatly affects the application of its aqueous solution and related dosage forms in various fields [14]. Therefore, it is of great significance to find more FA derivatives with similar functions.

Virtual screening (VS) is a common method for identifying new lead compounds from a compound database [15]. The ligand-based approach, which is used to discover promising candidates through structural optimization and property judgement. [16]. If the crystal structure of the target protein is known, a structure-based approach can be applied in research. Molecular docking is a basic way to evaluate the binding ability of compounds with proteins. Meanwhile, docking is able to identify the best combination position and then provides a score for each docked position. Furthermore, the use of a computer will undoubtedly be more conducive to the progress [17].

In this study, we aimed to search for novel ferulic acid derivatives with high anti-inflammation and antioxidant effects through virtual screening and biological experiments [16]. Initially, the flexible docking method was used to determine the action framework of FA, which was put into the InterBioScreen and Specs databases to find compounds with similar structures. Subsequently, the compounds were combined to build an FA derivatives database. Then, virtual screening was conducted through the CDOCKER method, and the drug likeness filters module was also applied to find compounds. The obtained results were further analyzed for free radical scavenging ability, while flow cytometry and microscopic observation of fluorescent staining were used to detect ROS. An MTT assay, ELISA and Western blotting were also employed to evaluate the potency of FA derivatives from different aspects.

## 2. Results

### 2.1. Virtual Screening

**Figure 1:**
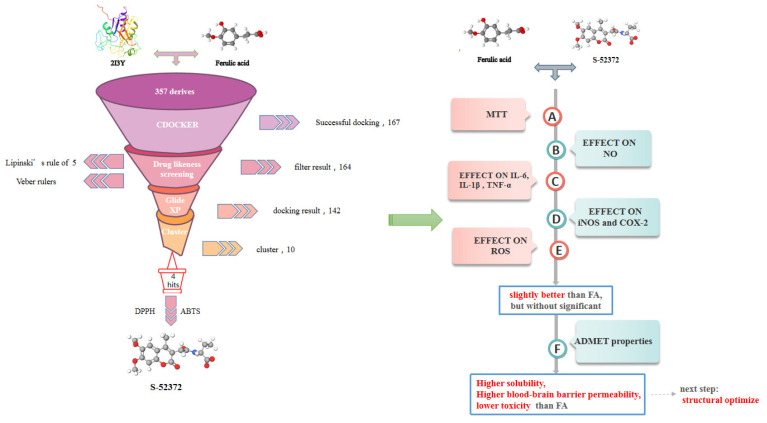
The whole process in this study.

**Figure 2:**
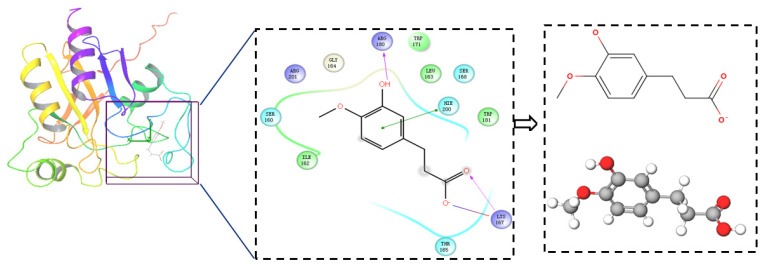
The docking results of FA with the active site of 2I3Y and the determined skeleton structure (green means Pi–Pi stacking, pink means H-bond, and the transition color means salt bridge).

From the docking results, we can see that all of the branches of the FA structure are important, so we decided to put the whole structure of FA into the InterBioScreen and Specs databases to search for FA derivatives with a similarity higher than 70%. As a result, 357 compounds were obtained to build an FA derivative database. The virtual screening protocol used in this study is shown in Figure 1. By analyzing the docking results of ferulic acid with GPx, the main structural features to support its activity can be measured. A two-dimensional view of the docking results is shown in Figure 2, from where we can see that FA formed hydrophobic and salt bridge interactions with the key residues ARG180 and LYS167 by interacting with hydroxyl and carboxyl groups; meanwhile, the benzene ring formed Pi–Pi stacking with HIS200.

**Figure 3:**
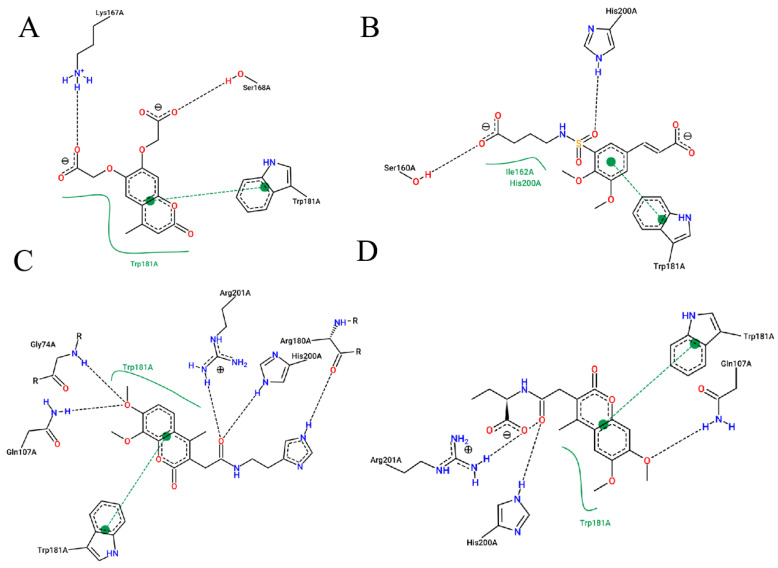
A floor plan view of the docking results, including (**A**) S-11593, (**B**) S-25207, (**C**) S-69904, and (**D**) S-52372.

As shown in Figure 1, after CDOCKER docking, 167 derivatives were retained. Then, Lipinski’s rule of five and Veber rules were further applied to filter the database, and 164 compounds were left for the succeeding experiments. The Glide XP module with higher docking precision was implemented, and 142 compounds were obtained. Then, via the hierarchical clustering of the canvas protocol, these compounds were clustered into 10 groups. Within these clusters, four compounds, shown in Table 1 and Figure 3, were chosen and purchased for subsequent experiments, because these four compounds have higher scoring values than FA, and the protonatable nitrogen and oxygen atoms can form key hydrogen bonds, salt bridges, or Pi–Pi interactions with the protein, thereby improving the stabilities of the docking complexes.

### 2.2. Extracellular Antioxidant Capacity Test

**Figure 4:**
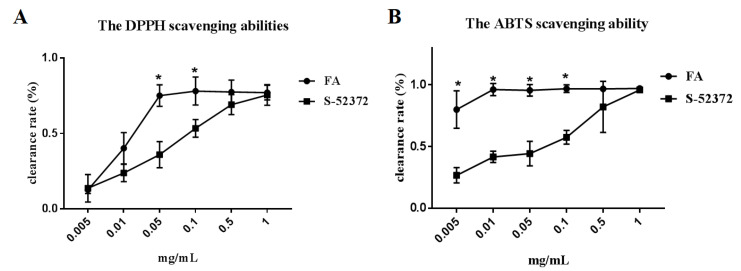
The graph of free radical scavenging ability. (**A**) The DPPH scavenging abilities of FA and its skeleton derivative, S-52372. (**B**) The ABTS scavenging ability of FA and its skeleton derivative S-52372. * *p* < 0.05 means FA compared with corresponding concentration of S-52372 group.

DPPH and ABTS radical scavenging ability experiments were carried out to verify their extracellular anti-oxidation effects. The results indicated that all four compounds had certain antioxidant capacities in the biochemical experiments, especially compound S-52372, which has the ability to scavenge free radicals in a concentration-dependent manner (as shown in Figure 4A,B), showing similar scavenge ability to FA at 1 mg/mL. Therefore, we decided to choose S-52372 to enter into the cell experiments to further investigate its anti-inflammatory and potential antioxidant activities.

Comparing the interaction modes of FA/S-52372 with target protein, the phenolic hydroxyl group of FA is very important in the interaction of FA and the target protein, as shown in Figure 2. There exist three hydrogen bonds, one formed between the phenolic hydroxyl group and ARG180, and the other two between the carboxyl group and LYS167. As for the compound S-52372, though without the phenolic hydroxyl group as FA, it can form three different hydrogen bonds with the protein, as shown in Figure 3D. Furthermore, a π–π interaction appeared between S-52372 and TRP181, which could further stable the binding system.

### 2.3. Cytotoxicity Measurement of FA and Its Derivative

**Figure 5:**
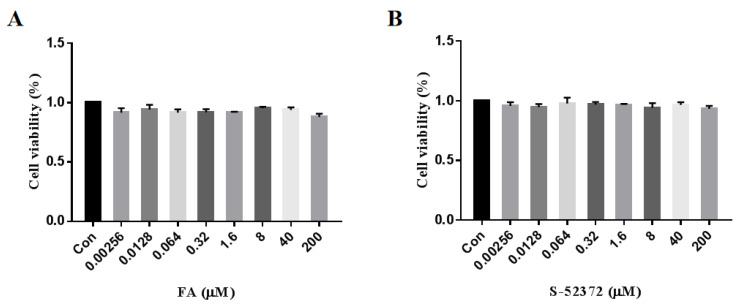
Cytotoxicity of ferulic acid and its derivative, S-52372, on RAW264.7 cells. RAW264.7 cells were treated with ferulic acid and S-52372 for 24 h, and an MTT assay was carried to detect the cell viability. (**A**) Cytotoxicity of ferulic acid on RAW264.7 cells. (**B**) Cytotoxicity of S-52372 on RAW264.7 cells. Each bar illustrates the average ± standard deviation (SD) counted from three experiments.

An MTT experiment was performed to ascertain whether ferulic acid and its derivative, S-52372, have cytotoxicity on RAW264.7 cells. As shown in Figure 5, the treatment with ferulic acid and S-52372 through concentrations from 0.00256 to 200 μmol/L for 24 h did not decrease the cell viability compared to the control group. Hence, these two compounds do not have obvious killing or poisoning effects on RAW264.7 cells in this concentration range, meaning they could be used in the succeeding steps. In order to study their anti-inflammatory effects, we chose a concentration range of 0.0128 to 8 μmol/L to preliminarily verify the anti-inflammatory properties by using the Griess method.

### 2.4. Effects on the Suppression of NO by FA and its Derivative

**Figure 6:**
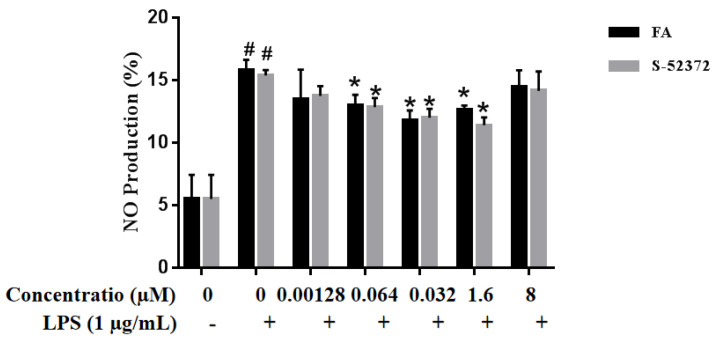
Inhibition of the FA and S-52372 on LPS-induced nitric oxide (NO) in RAW 264.7 macrophages. Each bar illustrates the average ± standard deviation (SD) counted from three experiments. * *p* < 0.05 compared to the only LPS−treated group; ^#^ *p* < 0.05 compared to the control group.

The RAW264.7 cells were immersed at various concentrations of FA and S-52372 at the concentration range from 0.0128 to 8 μmol/L induced with LPS (1 μg/mL). The obtained results showed that the NO concentration released by RAW264.7 cells increased significantly when added to with LPS compared to the control group without LPS, and the treatment with FA and S-52372 decreased the LPS-induced NO production. As shown in Figure 6, the two compounds showed a similar inhibitory trend on NO. The inhibitory effects are satisfactory, especially in the concentration range of 0.0128 to 1.6 μmol/L. With the decrease in compound concentrations, the inhibitory effects on NO production slightly decreased. At the concentration of 1.6 μmol/L, they showed a relatively good inhibitory effect and we found that S-52372 has a similar inhibitory effect to ferulic acid. Therefore, in order to facilitate the next experiment, we chose 0.064, 0.32, and 1.6 μmol/L for subsequent experiment research.

### 2.5. Inhibitory Effect of FA and S-52372 on IL-6, IL-1β, and TNF-α Production

**Figure 7:**
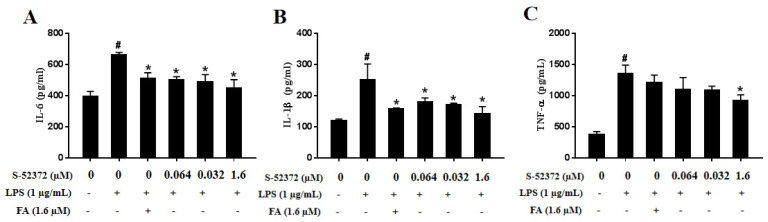
Effects of FA and S-52372 on TNF-α, IL-1β, and IL-6 induced by LPS. In addition to an untreated control sample, RAW264.7 cells were pretreated with 1.6 μmol/L of FA as a positive control to compare to RAW264.7 cells pretreated with with S-52372 at 1.6, 0.32, and 0.064 µM for 1.5 h, and then treated all groups with 1 µg/mL of LPS, except the control group, at 37 °C for 24 h. The levels of (**A**) TNF-α, (**B**) IL-1β, and (**C**) IL-6 in the culture supernatants of RAW264.7 macrophages were then detected by ELISA. Each bar represents the mean ± standard deviation (SD) calculated from three independent experiments. * *p* < 0.05 compared to only LPS−treated group; ^#^ *p* < 0.05 compared to the control group.

Aiming to exploe the suppression effect of FA and S-52372 on IL-6, IL-1β, and TNF-α production, we added the compounds to the cells that had been handed with LPS for 1.5 h in advance, and collected the cell supernatant after 24 h for centrifugation to conduct ELISA detection of different factors. FA proved to own good antioxidant and anti-inflammatory activities, so in the subsequent experiments, we selected the maximum concentration (1.6 μmol/L) of FA as a positive control, and then compared it to the screened derivate, S-52372, to obtain the corresponding trend and conclusion. As shown in Figure 7, compared to the unstimulated cells of the control group, the levels of IL-6, IL-1β, and TNF-α increased significantly in the LPS−induced cells of the model group. After adding FA and S-52372, the release of inflammatory factors produced by macrophages was inhibited to varying degrees. From Figure 7A, we can see that S-52372 displays an almost similar effective inhibitory effect on IL-6 production to ferulic acid (1.6 μmol/L). In addition, the derivative S-52372 also had a relatively concentration-dependent characteristic of inhibiting the release of IL-6, which is consistent with the previous NO results. For IL-1β and TNF-α production, as shown in Figure 7B,C, the addition of FA and S-52372 revealed a similar effect to IL-6.

### 2.6. Effect of FA and S-52372 on LPS−Induced Protein Expression of iNOS and COX-2

**Figure 8:**
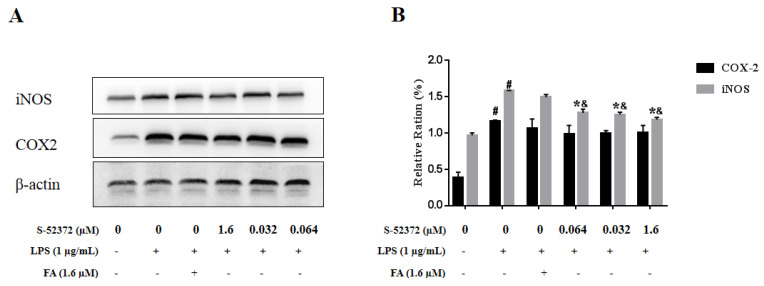
Ferulic acid and S-52372 decreased LPS−induced protein expressions of iNOS and COX-2. The protein expression of iNOS and COX-2 was detected by Western blot analysis. (**A**) iNOS and COX-2 protein expression levels. (**B**) Relative ration analysis. In addition to an untreated control sample, RAW264.7 cells were pretreated with 1.6 μmol/L of FA as a positive control to compare with RAW264.7 cells pretreated with with S-52372 at 1.6, 0.32, and 0.064 µM for 1.5 h, and then all groups were treated with 1 µg/mL of LPS, except the control group, at 37 °C for 24 h. Each bar represents the mean ± standard deviation (SD) calculated from three independent experiments. * *p* < 0.05 compared to the only LPS−treated group; ^#^ *p* < 0.05 compared to the control group; ^&^ *p* < 0.05 compared to the FA group.

In order to evaluate the effect of FA and S-52372 on proinflammatory mediators, the protein expressions of iNOS and COX-2 were determined. Figure 8 shows that S-52372 could reduce the expression of iNOS and COX-2 protein compared to the only LPS−treated model group; at the same time, S-52375 showed a significantl suppression effect at 1.6, 0.32, and 0.064 µM compared to FA (1.6 μmol/L). For the protein COX-2, the ability of derivative S-52372 to inhibit its expression was not different to FA in the selected concentration range, with almost no suppression effect.

### 2.7. Inhibitory Effect of FA and S-52372 on the ROS Production of LPS-induced RAW264.7 Cells

**Figure 9:**
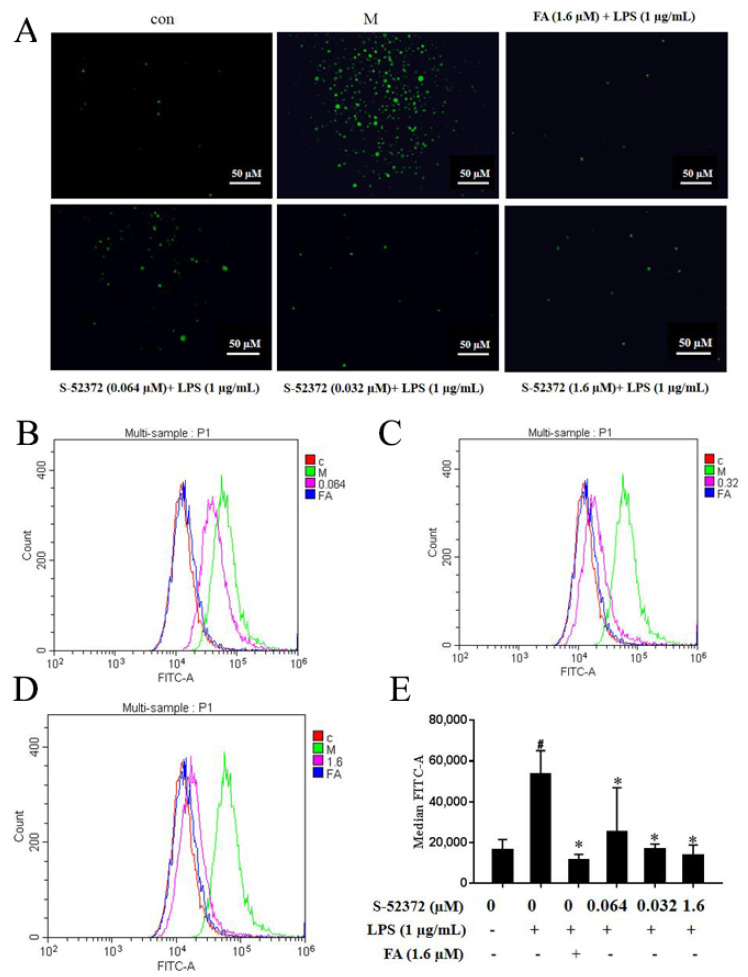
Ferulic acid and its derivative, S-52372, inhibited LPS−induced intracellular ROS production. In addition to an untreated control sample, RAW264.7 cells were pretreated with 1.6 μmol/L of FA as a positive control to compare with RAW264.7 cells pretreated with with S-52372 at 1.6, 0.32, and 0.064 µM for 1.5 h, and then all groups were treated with 1 µg/mL of LPS, except the control group, at 37 °C for 24 h. (**A**) ROS fluorescence images of FA (1.6 µM) and S-52372 (1.6, 0.32, and 0.064 µM). (**B**) ROS production at 0.064 µM of S-52372 to compare with FA at 1.6 µM. (**C**) ROS production at 0.32 µM of S-52372 to compare with FA at 1.6 µM. (**D**) ROS production at 1.6 µM of S-52372 to compare with FA at 1.6 µM. The results were presented in the above superposition of the fluorescence spectra of all groups. (**E**) Relative fluorescence intensity analysis. * *p* < 0.05 compared to the only LPS-treated group; ^#^ *p* < 0.05 compared to the control group.

To evaluate the effect of FA and S-52372 on the oxidative stress induced by LPS, fluorescence staining and flow cytometry were used to examine their effects on intracellular ROS production. Figure 9 shows that after LPS stimulation alone, the release of ROS was strongly stimulated, as shown by the green normal distribution curve. After adding ferulic acid, the normal distribution curve had a significant shift in cytofluorogram to the left and almost reached a coincidence with the control group. Compared to the model group, the distribution of the S-52372 also had an obvious shift to the left, indicating that it had an inhibitory effect on the production of reactive oxygen species in the cells. Meanwhile, the inhibitory effect gradually weakened as the concentration decreased. Additionally, a significant decline in the average fluorescence intensity was detected through fluorescence staining. Compared to the model group, the image also indicated a sharp decrease in intracellular ROS after treatment with S-52372, while the average fluorescence intensity was significantly changed in the concentration range of 0.064 to 1.6 µM (*p* < 0.05) for S-52372. Therefore, FA and its derivative, S-52372, could reduce the LPS−induced production of intracellular ROS.

### 2.8. Computer-Aided Prediction of the Related Properties of S-52372 and FA

After the completion of a series of experiments, the experimental results show that S-52372 and FA have comparable antioxidant and anti-inflammatory effects, and its effect is a little better than FA in some cases. Then, we used the computer’s drug prediction function to evaluate and compare the relative properties of these two compounds. The results show that both S-52372 and FA could be dissolved in water and located at −4.1 < log(Sw) < −2.1, and S-52372 has better water solubility than FA for the higher log(Sw) of S-52372. As for the blood–brain barrier level, it can be predicted that S-52372 has better blood–brain barrier permeability than FA. In the toxicity prediction, we can see that FA could reach the LD_50_ of the rat acute toxicity dose at 1.4314 moL/kg, while the LD_50_ of S-52372 was higher, showing that S-52372 is less toxic than FA. Their predicted values of cell permeability were not much different, both being relatively good. All results as shown in Table 2.

## 3. Discussion

Ferulic acid (FA) is a polyphenolic compound and is widely used to scavenge free radicals and anti-oxidative and anti-inflammatory factors. It has been proposed that FA possesses the ability to exert effort on cancers and cardiovascular disease [18]. However, there are related literature reports on the stability of ferulic acid being affected by temperature, pH, and other influencing factors, which greatly affects the application of its aqueous solution and related dosage forms in various fields [19]. Therefore, it is of great significance to find compounds with similar structures and the same functions with ferulic acid. In this study, we found a compound from a natural database, S-52372, which has a similar skeleton to ferulic acid and was obtained through virtual screening.

Oxidative stress is the main pathway for producing free radicals involving ROS. The increased concentrations of ROS in organisms has a close relationship with the development of diseases [20]. Researchers have discovered that natural sources could prevent or reduce oxidative stress. Some researchers have clearly shown that natural sources such as ferulic acid can slow down the oxidative stress process [21]. The free radical scavenging capacity was measured in the cell-free system using DPPH and ABTS assays to evaluate its extracellular antioxidant capacity [20]. The experimental results showed that as the concentration of S-52372 increases, the scavenging ability of free radicals also increases, and at the highest concentration, it shows a scavenging rate similar to that of FA.

Macrophages, which can quickly respond to lipopolysaccharide (LPS) stimulation, are often used as cell modules in research related to inflammation. Macrophages can be stimulated by the outer membrane of Gram-negative bacteria, and then active inflammation pathogens [22]. LPS triggers inflammatory processes mainly through the toll-like receptor 4 (TLR4) signaling pathway, then causes more sophisticated biological responses involving the production of pro-inflammatory mediators [23]. Inflammatory disorders are also closely related to NF-κB, which triggers a typical signal pathway. A family of inhibitory proteins (IκBs) can manage inactive NF-κB. The IκB kinase complex (IKK) is also involved in the NF-κB pathway. IKK leads to the phosphorylation and degradation of IκB. In the following steps, NF-κB is activated and stimulates the transcription and expression of pro-inflammatory genes, finally leading to high concentrations of all kinds of factors such as IL-6, TNF-α, and IL-1β [24].

The anti-inflammatory activities of FA and S-52372 were determined in intracellular experiments on an LPS-induced macrophage RAW264.7 cell model to explore their inflammatory inhibition abilities. We used the ELISA method to detect the inhibitory effects of the compounds on the production of pro-inflammatory factors, as well as WB methods to measure the effects of the compounds on the protein expressions of iNOS and COX-2. The antioxidant activity of S-52372 was determined through fluorescence staining and flow cytometry. The functional assays demonstrated that both FA and S-52372 can limit ROS formation in the presence of inflammatory macrophages and can reduce the release of NO, IL-6, TNF-α, IL-1β, iNOX, and COX-2 by lipopolysaccharide-treated macrophages. In the computer prediction, we found that S-52372 owns better water solubility and blood–brain barrier permeability than FA.

## 4. Materials and Methods

### 4.1. Materials and Chemicals

RAW264.7 cells were bought from the Chinese Academy of Science (Chinese Academy of Science, Beijing, China). Dulbecco Minimum Essential Medium (DMEM) culture medium, penicillin streptomycin combination, and trypsin (0.25%) were purchased from HyClone (HyClone, Los Angeles, UT, USA). Fetal bovine serum (FBS) was bought from Gemini (Gemini, Woodland, CA, USA). NO kits were obtained from Nanjing Jiancheng Bioengineering Institute (Nanjing Jiancheng Bioengineering Institute, Nanjing, China). TNF-α, IL-1β, and IL-6 kits were obtained from Boster (Boster, Wuhan, China). Ferulic acid and ferulic derivatives were purchased from TargetMol (TargetMol, Shanghai, China). LPS was purchased from Sigma-Aldrich (Sigma-Aldrich, St. Louis, MO, USA). 1,1-Diphenyl-2-picrylhydrazyl radica (DPPH), 2′,7′-dichloro-flfluorescin (DCFH-DA), and Thiazolyl tetrazolium (MTT) were purchased from Solarbio Science & Technology (Solarbio, Beijing, China). RAW264.7 cells were cultivated in a 5% CO_2_ incubator at 37 °C, and the culture medium was mixed with 13% fetal bovine serum and 1% penicillin–streptomycin in DMEM culture medium.

### 4.2. Computer-Aided Virtual Screening

Virtual screening, a reliable and relatively inexpensive technique for potential discovery of compounds, has been widely used in drug discovery [25].

#### 4.2.1. Obtaining FA Derivatives and Primary Docking Screening

Glutathione peroxidase (GPx), an antioxidant enzyme, is a typical oxidative stress marker. By measuring the change in GPx, we can learn about the variation in the oxidation system after adding FA [26]. We downloaded the glutathione peroxidase (GPx) crystal structure (PDB entry: 2I3Y) from the RCSB Protein Data Bank database [27], and the structure was pretreated by the prepare protein protocol through Accelrys Discovery Studio 2.5 software [28]. The active binding site was defined as being the same as the small molecule presented in 2I3Y. The structure of ferulic acid was prepared using Prepare Ligands tools. Then, the flexible docking protocol was implemented to explore the interaction between the active ingredient and the target protein, and evaluation of the results was carried out according to the interaction mode (hydrogen bonding, van der Waals force, etc.). Based on the results, the key ferulic acid structural skeleton was selected, which was put into the InterBioScreen and Specs databases to search for FA derivatives with 70% structural similarity. The obtained compounds were combined to build an FA derivatives database, and then fast docking was implemented using the CDOCKER protocol to explore the interaction between the compounds and the target protein. Evaluation of the results was carried out according to the docking score and the interaction mode.

#### 4.2.2. Drug Likeness Evaluation

Compounds were screened using Lipinski’s rule of five. If a compound owning more than five hydrogen bond donors, ten hydrogen bond acceptors, a molecular weight (MWT) >500, and a calculated LogP (CLogP) >5, it may show weaker absorption ability and instability [29]. Based on those properties, compounds such as these were excluded. After this, other compounds were filtered by the Veber rules, which includes 10 or fewer rotatable bonds and a polar surface area equal to or <140 Å^2^ [30].

#### 4.2.3. High Precision Screening

Glide XP (extra Precision) docking was conducted to further screen FA derivatives. The compounds were added to with an OPLS_2005 force field, and then shifted to the charged states at pH 7.0 ± 2 in Schrödinger.

### 4.3. Antioxidant Capacity Evaluation

After virtual screening, DPPH and ABTS free radical scavenging experiments were conducted to evaluate the corresponding antioxidant activity in the biochemical experiments [31]. First, a series of sample solutions were prepared, and then the control, sample, and blank groups were set up as required to carry out DPPH and ABTS radical scavenging assays.

#### 4.3.1. DPPH Radical Scavenging Activity

A total of 40 μL of each sample of a series of concentrations was brewed with 160 μL of a 0.1 mM/L absolute ethyl alcohol solution of DPPH, and the solution was placed for 30 min in an environment without light. The OD was measured at 517 nm and then the average value was calculated, after which the DPPH clearance rate of each concentration was obtained to make a line graph.

Clearance rate = (1 − (A sample − A blank)/A control) ∗ 100%

#### 4.3.2. ABTS Radical Scavenging Activity

An equal amount of ABTS stock solution was mixed with potassium persulfate stock solution and kept away from light for 12–16 h. Then, the mixture was diluted with pH 7.4 PBS to the ABTS working solution with an absorbance of 0.7 ± 0.05 at 405 nm. Next, 10 μL of each sample with of a series of concentrations was fused with 200 μL of the ABTS working solution, and the mixed solution was reacted for 6 min in the environment without light. The OD was measured at 517 nm and then the average data were calculated, after which the ABTS clearance rate of each concentration was also obtained to make a line graph.

Clearance rate = (1 − (A sample − A blank)/A control) ∗ 100%

### 4.4. Cell Culture and Stimulation

The RAW264.7 macrophage line was maintained in DMEM supplemented with 13% fetal bovine serum (FBS) and 1% penicillin–streptomycin (P/S) at 37 °C in a 5% CO_2_-humidified air environment. The cells were handled in advance with corresponding compounds for 2 h in serum-free media, then LPS (1 µg/mL) was added to stimulate cells.

#### 4.4.1. Cell Viability Assay

RAW264.7 cells were seeded in a 96-well plate overnight at a density of 5000 cells in a single well to make sure that the cells adhered to the plate. After observing complete adhesion, 24 h later, the original medium was aspirated and 150 µL of compound solutions of different concentrations was add. After incubation with the compound for 24 h, 15 µL of MTT (5 mg/mL) was added to each well and incubated in an incubator for 4 h. Then, 150 μL of the triple solution was added to each well to dissolve the formazan crystals overnight (the solution was made up of 10% SDS, 5% isobutanol, and 0.01 mol/L of HCl aqueous solution). The next day, a microplate reader was used to measure the absorbance at 570 nm.

#### 4.4.2. Measurement of NO

RAW264.7 cells were seeded at 5000 cells per well and kept in 96-well plates. After incubation overnight at 37 °C, ferulic acid and its derivative were added to the each well at the decided concentrations for 1.5 h in medium before adding LPS (1 µg/mL). After incubation again for another 24 h after adding the compounds, NO production was measured by collecting the supernatants using a nitrate/nitrite assay kit. Then, the OD figure was measured at an optical density of 540 nm.

#### 4.4.3. Determinations of IL-6, TNF-α, and IL-1β Levels

RAW264.7 cells were plated in a 24-well plate and kept for 24 h at 37 °C at 2 × 10 ^5^ cells/well. The cells were treated with ferulic acid and its derivative at the decided concentrations for 1.5 h in medium before adding LPS (1 µg/mL). Twenty-four hours later, the supernatants were collected. The expressed levels of IL-6, TNF-α, and IL-1β were measured by ELISA kits (Boster, Wuhan, China) according to the instructions of the manufacturer.

### 4.5. Western Blot Analysis

The cells were treated with ferulic acid and its derivative at the decided concentrations for 1.5 h in medium before adding LPS (1 µg/mL); then, the cells were collected at the same time. Phosphate-buffered saline (PBS) was used to clean the cells and blow the cells from the bottom of the medium. Then, cells were finally obtained by centrifugal collection at 1500 rpm for 6 min at 4 °C. After adding the cell lysate, the mixture was placed on the ice and shaken at a constant speed for 30 min, followed by centrifugation at 14,000× *g* for 15 min at 4 °C. The protein concentrations were determined by a Bradford protein assay. After adding the sample to the groove, the related process of protein separation and membrane transfer were carried out, then the sample reacted with the antibody, at the end proteins fixed on the membrane. Finally, subsequent visualization of antibody binding was carried out with enhanced chemiluminescence and the obtained images were analyzed by ImageJ software.

### 4.6. Determination of Intracellular ROS Content

The pre-processed RAW264.7 cells were plated in the six-well plate and then collected with the medium, then incubated with the fluorescent probe 2′,7′-dichlorodihydrofluorescein diacetate (DCFH-DA, 10 µM) at 37 °C for 30 min. PBS was used three times to remove the dye. Finally, the outcome was verified by flow cytometry. We observed the intensity of fluorescence under a fluorescence microscope.

### 4.7. Prediction of ADMET Properties

In Tools Explorer, we selected the Small Molecules option, calculated the molecular properties, and clicked on the ADMET Descriptors option to open the ADMET Descriptors dialog box. Then, we selected the grid to the right of Input Ligands and chose pk-test: All in the drop-down list, before selecting all small molecule compounds. The ADMET Descriptors parameter selected the default setting, that is, all ADMET properties were selected. This operation calculated all ADMET properties of all molecules.

### 4.8. Statistical Analysis

All of the experiments were conducted three times. All the data were expressed as the average ± standard deviation (SD). Figure significance was measured by one-way ANOVA analysis. *p* < 0.05 indicates a significant difference.

## 5. Conclusions

In this study, we successfully found the compound S-52372 from natural databases by combing computer-aided virtual screening and an experimental protocol, which possess similar bioactivities to FA in terms of anti-inflammatory and antioxidant activities in RAW264.7 cells stimulated by LPS, through slightly hindering the ROS production and impacting the expressions of inflammatory mediators, including iNOS, COX-2, TNF-α, and IL-6. In the computer prediction, we found that S-52372 has better water solubility and blood–brain barrier permeability and lower toxicity than FA. Thus, this compound deserves further research for potential usage for skin anti-inflammation and anti-oxidation.

## Figures and Tables

**Table 1:** The obtained potential derives by virtual screening.

No.	Database	Structure	Docking Score
FA		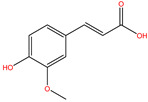	−4.608
S-69904	InterBioScreen	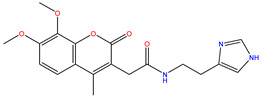	−4.985
S-52372	InterBioScreen	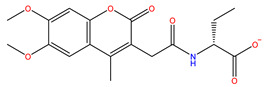	−5.024
S-11593	InterBioScreen	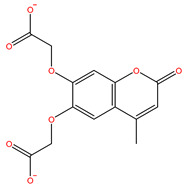	−5.841
S-25207	InterBioScreen	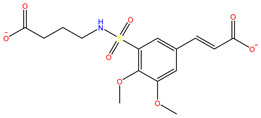	−4.379

**Table 2:** Computer prediction of the related properties of S-52372 and FA.

Model	FA	s-52372	FA	s-52372
**Absorbtion**				
Blood–Brain Barrier	BBB-	BBB-	0.5305	0.9526
Human Intestinal Absorption	HIA+	HIA+	0.9614	0.8187
Aqueous Solubility	−2.4766	−1.5911	LogS	
Caco-2 Permeability	Caco2+	Caco2-	0.7183	0.7275
**Metabolism**				
CYP450 2C9 Substrate	Non-substrate	Non-substrate	0.7464	0.8111
CYP450 2D6 Substrate	Non-substrate	Non-substrate	0.8922	0.8404
CYP450 1A2 Inhibitor	Non-inhibitor	Non-inhibitor	0.7513	0.6462
CYP450 2D6 Inhibitor	Non-inhibitor	Non-inhibitor	0.9588	0.9165
CYP450 3A4 Inhibitor	Non-inhibitor	Non-inhibitor	0.924	0.8916
**Toxicity**				
AMES Toxicity	Non-AMES-toxic	Non-AMES-toxic	0.9132	0.6156
Carcinogens	Non-carcinogens	Non-carcinogens	0.9076	0.9523
Acute Oral Toxicity	IV	III	0.6265	0.5929
Rat Acute Toxicity	1.4314	2.8767	LD_50_, moL/kg	

## Data Availability

Data is contained within the article.

## References

[1] Di Meglio P., Perera G.K., Nestle F.O. (2011). The multitasking organ: Recent insights into skin immune function. Immunity.

[2] Ghezzi P., Floridi L., Boraschi D., Cuadrado A., Manda G., Levic S., D’Acquisto F., Hamilton A., Athersuch T.J., Selley L. (2018). Oxidative Stress and Inflammation Induced by Environmental and Psychological Stressors: A Biomarker Perspective. Antioxid. Redox Signal.

[3] Sen C.K., Roy S. (2008). Redox signals in wound healing. Biochim. Biophys. Acta.

[4] Akashi S., Saitoh S., Wakabayashi Y., Kikuchi T., Takamura N., Nagai Y., Kusumoto Y., Fukase K., Kusumoto S., Adachi Y. (2003). Lipopolysaccharide interaction with cell surface Toll-like receptor 4-MD-2: Higher affinity than that with MD-2 or CD14. J. Exp. Med..

[5] Zou J., Feng D., Ling W.H., Duan R.D. (2013). Lycopene suppresses proinflammatory response in lipopolysaccharide-stimulated macrophages by inhibiting ROS-induced trafficking of TLR4 to lipid raft-like domains. J. Nutr. Biochem..

[6] Pizzino G., Irrera N., Cucinotta M., Pallio G., Mannino F., Arcoraci V., Squadrito F., Altavilla D., Bitto A. (2017). Oxidative Stress: Harms and Benefits for Human Health. Oxid. Med. Cell Longev..

[7] Moalem G., Tracey D.J. (2006). Immune and inflammatory mechanisms in neuropathic pain. Brain Res. Rev..

[8] Lee S.B., Lee W.S., Shin J.S., Jang D.S., Lee K.T. (2017). Xanthotoxin suppresses LPS-induced expression of iNOS, COX-2, TNF-alpha, and IL-6 via AP-1, NF-kappaB, and JAK-STAT inactivation in RAW 264.7 macrophages. Int. Immunopharmacol..

[9] Abotaleb M., Liskova A., Kubatka P., Busselberg D. (2020). Therapeutic Potential of Plant Phenolic Acids in the Treatment of Cancer. Biomolecules.

[10] Karthikeyan S., Kanimozhi G., Prasad N.R., Mahalakshmi R. (2011). Radiosensitizing effect of ferulic acid on human cervical carcinoma cells in vitro. Toxicol. In Vitro.

[11] Kwon M.Y., Kim S.M., Park J., Lee J., Cho H., Lee H., Jeon C., Park J.H., Han I.O. (2019). A caffeic acid-ferulic acid hybrid compound attenuates lipopolysaccharide-mediated inflammation in BV2 and RAW264.7 cells. Biochem. Biophys. Res. Commun..

[12] Sultana R., Ravagna A., Mohmmad-Abdul H., Calabrese V., Butterfield D.A. (2005). Ferulic acid ethyl ester protects neurons against amyloid beta-peptide(1-42)-induced oxidative stress and neurotoxicity: Relationship to antioxidant activity. J. Neurochem..

[13] Panneerselvam L., Subbiah K., Arumugam A., Senapathy J.G. (2013). Ferulic acid modulates fluoride-induced oxidative hepatotoxicity in male Wistar rats. Biol. Trace Elem. Res..

[14] Kumar N., Pruthi V. (2014). Potential applications of ferulic acid from natural sources. Biotechnol. Rep..

[15] Costa E., Cosme F., Jordão A.M. (2014). Anthocyanin profile and antioxidant activity from grape varieties cultivated in two Portuguese wine regions. J. Int. Des Sci..

[16] Kitchen D.B., Decornez H., Furr J.R., Bajorath J. (2004). Docking and scoring in virtual screening for drug discovery: Methods and applications. Nat. Rev. Drug. Discov..

[17] Willett P. (2006). Similarity-based virtual screening using 2D fingerprints. Drug Discov. Today.

[18] Szulc-Kielbik I., Kielbik M., Klink M. (2017). Ferulic acid but not alpha-lipoic acid effectively protects THP-1-derived macrophages from oxidant and pro-inflammatory response to LPS. Immunopharmacol. Immunotoxicol..

[19] Thyagaraju B.M. (2008). Muralidhara, Ferulic acid supplements abrogate oxidative impairments in liver and testis in the streptozotocin-diabetic rat. Zoolog. Sci..

[20] Sridhar K., Charles A.L. (2019). In vitro antioxidant activity of Kyoho grape extracts in DPPH and ABTS assays: Estimation methods for EC50 using advanced statistical programs. Food Chem..

[21] Burin V.M., Ferreira-Lima N.E., Panceri C.P. (2014). Bioactive 530 compounds and antioxidant activity of Vitis vinifera and Vitis labrusca grapes: 531 Evaluation of different extraction methods. Microchem. J..

[22] Beutler B., Jiang Z., Georgel P., Crozat K., Croker B., Rutschmann S., Du X., Hoebe K. (2006). Genetic analysis of host resistance: Toll-like receptor signaling and immunity at large. Annu Rev. Immunol..

[23] Palsson-McDermott E.M., O’Neill L.A. (2004). Signal transduction by the lipopolysaccharide receptor, Toll-like receptor-4. Immunology.

[24] Piazza M., Calabrese V., Baruffa C., Gioannini T., Weiss J., Peri F. (2010). The cationic amphiphile 3,4-bis(tetradecyloxy)benzylamine inhibits LPS signaling by competing with endotoxin for CD14 binding. Biochem Pharmacol.

[25] Han W., Shi Y., Su J., Zhao Z., Wang X., Li J., Liu H. (2020). Virtual Screening and Bioactivity Evaluation of Novel Androgen Receptor Antagonists From Anti-PCa Traditional Chinese Medicine Prescriptions. Front Chem..

[26] Margis R., Dunand C., Teixeira F.K., Margis-Pinheiro M. (2008). Glutathione peroxidase Family—An evolutionary overview. FEBS J..

[27] Wang Y., Branicky R., Noe A., Hekimi S. (2018). Superoxide dismutases: Dual roles in controlling ROS damage and regulating ROS signaling. J. Cell Biol..

[28] Accelrys (2010). Accelrys Discovery Studio, Version 2.5.

[29] Lipinski C.A., Lombardo F., Dominy B.W., Feeney P.J. (2001). Experimental and computational approaches to estimate solubility and permeability in drug discovery and development settings. Drug Deliv..

[30] Veber D.F., Johnson S.R., Cheng H.Y., Smith B.R., Ward K.W., Kopple K.D. (2002). Molecular properties that inflfluence the oral bioavailability of drug candidates. Med. Chem..

[31] Kotora P., Sersen F., Filo J., Loos D., Gregan J., Gregan F. (2016). The Scavenging of DPPH, Galvinoxyl and ABTS Radicals by Imine Analogs of Resveratrol. Molecules.

